# The effect of a treatment switch to integrase Strand transfer inhibitor–based regimens on weight gain and other metabolic syndrome-related conditions

**DOI:** 10.1186/s12879-024-09120-7

**Published:** 2024-02-19

**Authors:** Omer Maman, Wiessam Abu Ahmad, Ofer Perzon, Keren Mahlab-Guri, Daniel Elbirt, Hila Elinav

**Affiliations:** 1 Medical School, Hebrew University, Jerusalem, Israel; 2 Imaging division, Radiology department, Sourasky medical center-Ichilov, Tel Aviv, Israel; 3 Braun School of Public Health and Community Medicine, Hebrew University, Jerusalem, Israel; 4 Internal medicine ward B, Hadassah Hebrew University Medical Center, Jerusalem, Israel; 5 Department of Allergy, Immunology and HIV, Kaplan Medical Center, Rehovot, Israel; 6 Faculty of Medicine, Hebrew University of Jerusalem, Jerusalem, Israel; 7 Hadassah AIDS Center, Department of Microbiology and Infectious Diseases, Hadassah Hebrew University Medical Center, Jerusalem, Israel

**Keywords:** INSTI, Weight gain, Switch, Hyperglycemia, Risk factors

## Abstract

**Objective:**

This study aimed to assess weight gain associated with treatment switching to INSTI-based regimens in people living with HIV (PLWH) and to determine whether it is accompanied by worsening features of hypertension, dyslipidemia, or hyperglycemia.

**Methods:**

In this two-center retrospective observational study, we assessed weight gain and metabolic features in PLWH who switched to an INSTI-based regimen (study group) as compared to patients who remained on a non-INSTI regimen (control group) over a 24-month follow-up period.

**Results:**

One-hundred seventy-four PLWH were included in the study group, and 175 were included in the control group. The study group gained 2.51 kg ± 0.31 (mean ± standard deviation) over the 2 years of follow-up, while the control group gained 1.1 ± 0.31 kg over the same time course (*p* < 0.001). INSTI treatment, Caucasian origin, and lower BMI were risk factors associated with excessive weight gain during the 2 years of follow-up. Among metabolic parameters, only glucose levels increased after initiating INSTI-based regimens, although limited to males of African origin (*p* = 0.009).

**Conclusions:**

We observed a mild weight gain after switching to INSTI-based regimens, with no major impact on metabolic parameters over 2 years of follow-up. Longer follow-up might be needed to observe the adverse metabolic effects of INSTI-based regimens. The impact on weight gain should be discussed with every patient before the treatment switch to ensure a balanced diet and physical activity to prevent excessive weight gain that might hamper compliance with ART.

## Introduction

Integrase inhibitors (Integrase Strand Transfer Inhibitor – INSTI) [1] revolutionized antiretroviral treatment. They were proven to achieve superior viral suppression as compared to protease inhibitors (PIs) and have an improved drug-drug interaction and safety profile, leading to their inclusion as core agents in most guidelines of antiretroviral treatment (ART) regimens [2–4]. Nevertheless, these drugs are not free from adverse effects. INSTIs are associated with side effects impacting the central nervous system, including headaches, insomnia, mood disturbances, abnormal dreaming, and dizziness [5]. Other adverse effects include nausea, diarrhea, rash, and increased serum creatinine levels. Some studies [6, 7] have suggested that weight gain may be a consequence of treatment with INSTI, while this was not observed in other studies [8]. In addition, there were substantial discrepancies in the extent of weight gain noted between different studies [9–15].

ART-associated weight gain is a known phenomenon commonly observed in the first year of antiretroviral treatment and is often ascribed to a general improvement of the patient’s condition, a term known as “return to health” [16–18]. There is evidence indicating that weight gain may also be an adverse effect of combined antiretroviral treatment (cART), which may exacerbate metabolic disturbances such as dyslipidemia and insulin resistance and contribute to the development of cardiovascular complications [18–22]. Additionally, drug-related weight gain may have a negative effect on patients’ compliance with their life-saving medical treatment.

In this study, we evaluated whether the transition to INSTI-based regimens leads to weight gain and worsening of metabolic syndrome-related conditions. To this end, we compared PLWH who were switched to INSTI-based regimens with those who continued treatment with non-INSTI-based regimens, namely non-nucleoside reverse transcriptase inhibitors (NNRTIs) and protease inhibitors (PIs). We further examined the effects exerted by different medications within the INSTIs family and the accompanying nucleoside reverse transcriptase inhibitors (NRTIs) on metabolic features in ART-treated PLWH, and studied whether demographic characteristics such as age, gender, or ethnicity play a role in INSTI-associated weight gain.

## Methods

This retrospective observational clinical study included adult PLWH treated with PI or NNRTI-based cART for at least a year in two HIV clinics in Israel. Two groups of PLWH were compared: PLWH who switched to an INSTI-based regimen and a control group that maintained a non INSTI-based ART regimen. Clinical parameters were retrieved from medical records at three different time points. “Time 0” was defined for participants in the study group as the day of treatment change to an INSTI-based regimen, after successfully being treated for at least 1 year with a non- INSTI-based regimen. In the control group, “Time 0” was defined as an arbitrary day, at least 1 year after being successfully treated with a non-INSTI based regimen. Additional data was collected at 12 ± 2 months and 24 ± 2 months after time 0. The parameters retrieved from the medical records included gender, origin, date of birth, weight (kg, at diagnosis, treatment initiation and study follow-up), height (cm), BMI (kg/m^2^), blood pressure (mmHg) and biochemical tests, including fasting triglycerides, total cholesterol, LDL, HDL, and fasting glucose levels (all in mg/dL). All parameters were evaluated during routine clinic follow-up visits at 0, 12 ± 2 months and 24 ± 2 months. HIV-related parameters were date of HIV diagnosis, date of treatment initiation, CD4 cell counts and HIV viral load (VL) at diagnosis, treatment initiation, and study follow-up (time 0, 12 ± 2 months and 24 ± 2 months), ART history and duration of viral suppression until time 0 (months). Patients were excluded from the study in cases of HBV or HCV cross-infections, opportunistic infections, hospitalization, or pregnancy during the period of follow-up.

### Statistical analysis

Patient characteristics were reported by descriptive statistics using all available information. Demographic data were analyzed by independent sample t-test or by Chi-square test (or likelihood ratio test, G-test) according to the scale of the variable, and descriptive statistics are given as mean (M) with standard deviation (SD) or as frequency (n) with percentage (%). Two-order interactions: study group*time and three-order interactions: risk factor (gender, origin or age)*study group*time were assessed by repeated measures mixed-effect models with a random intercept at the participant level,after adjusting for covariates. These models were followed by post-hoc analysis to test the differences in time between all levels of risk factors and study groups. The analysis was done using the Benjamini-Hochberg procedure to control the type I error. The models were adjusted for CD4 and BMI at index time, which were included as they were influential in the model. We also performed a multivariate analysis to identify risk factors for gaining excessive weight, defined as more than 5% weight gain during 2 years of follow-up. Variables included in the analysis were study group (control or switch), origin, gender, age, BMI and CD4 levels at index time (time 0). A sensitivity analysis introduced an alternative criterion for defining excessive weight gain: individuals in the highest quintile of the relative weight difference, either within a one-year follow-up or 2 years (gained more than 5.6 kg during 1 year of follow-up or 7.3 kg during 2 years). *P*-value < 0.05 was considered statistically significant. All reported *p*-values are two-tailed. Analysis was done with IBM SPSS STATISTICS version 25.0, Stata/SE version 15.0 (StataCorp) and R statistical software version 3.5.0 (R Project for Statistical Computing).

### Ethics

This research was approved by the Hadassah-Hebrew University Medical Center Institutional Review Board (IRB) (0259–19-HMO) and the Kaplan Medical Center ethics committee (KMC-0036-10). Due to the retrospective design of the research, a waiver of informed consent of the participants in the study was provided by both ethics committees.

## Results

Approximately two thousand files of PLWH were screened at two AIDS centers: Neve Or (Kaplan Medical Center, Rehovot, Israel) and the Hadassah AIDS Center (Hadassah - Hebrew University Medical Center, Jerusalem, Israel). Three hundred and forty nine PLWH were included: 174 participants were included in the study group and 175 in the control group. The two groups were comparable in age, gender, risk groups, time from HIV diagnosis to treatment initiation, CD4 T-cell counts, viral load, and weight at diagnosis and Time 0 (Table 1). BMI differed only at the time of HIV diagnosis but was comparable at Time 0. Viral load suppression time until Time 0 was longer in the study group (71 months vs. 56 months in the control group, *p* = 0.0028). Almost half of the participants (84/174, 48.3%) who switched to INSTI were prescribed Dolutegravir. Tenofovir disoproxil fumarate/Emtricitabine (TDF/FTC) was the most prevalent backbone used in the control and study groups before the treatment switch (295/349, 84.5%). Usage of TDF/FTC as a backbone decreased to 54% following the switch, as patients were switched to INSTI regimens combined with abacavir/lamivudine (ABC/3TC) or Tenofovir alafenamide/Emtricitabine (TAF/FTC) (Table 1).

**Table 1:** Basic characteristics

Characteristic	Study Group (***n*** = 174)		Control Group (***n*** = 175)	*P*-value
**Demographics**				
Mean age (years)	47.3 ± 11.9		46.5 ± 10.9	0.491
**Age (subgroups):**				0.770
18–41 y.o.	60 (34.5%)		66 (37.7%)	
42–51 y.o.	60 (34.5%)		54 (30.9%)	
≥ 52 y.o.	54 (31%)		55 (31.4%)	
Gender - Male	91 (52.3%)		105 (60.0%)	0.132
Origin -African descendant	122 (70.1%)		117 (66.9%)	0.490
**HIV Parameters at diagnosis**				
Viral load (copies/ml)	323,622 ± 702,463		837,396 ± 5,915,115	0.313
CD4 (cells/μl)	239 ± 178		272 ± 234	0.187
Nadir CD4 (cells/μl)	147 ± 94		159 ± 125	0.358
Weight (kg)	60.4 ± 10.5		63.4 ± 13.2	0.071
BMI (kg/m^2^)	21.9 ± 3.2		22.9 ± 3.9	0.045
**Time between HIV diagnosis and ART initiation (years)**	2.4 ± 3.08		1.86 ± 3.03	0.175
**Time between diagnosis and time 0 (years)**	10.48 ± 5.4		7.86 ± 4.38	< 0.001
**Treatment duration before time 0 (years)**	8.03 ± 4.79		6.10 ± 3.86	< 0.001
**Viral suppression duration before time 0**	70.0 ± 50.9		56.0 ± 4.3	0.0028
**Parameters at time 0**				
Viral load (copies/ml)	9539 ± 80,479		503 ± 6292	0.14
Patients with viral load ≤50	166 (95.4%)		162 (92.6%)	0.26
CD4 (cells/μl)	533 ± 248		518 ± 258	0.59
Weight (kg)	66.7 ± 12		68.7 ± 13.0	0.136
BMI (kg/m^2^)	24.4 ± 3.7		24.8 ± 3.9	0.3
Mean blood pressure (mmHg)	88 ± 10		90 ± 10	0.2
Total cholesterol (mg/dL)	187 ± 43		190 ± 43	0.44
LDL (mg/dL)	113 ± 44		115 ± 34	0.8
HDL (mg/dL)	47 ± 14		48.3 ± 13.5	0.4
Triglycerides (mg/dL)	154 ± 96		155 ± 143	0.939
Glucose (mg/dL)	97.7 ± 28.2		97.1 ± 28.0	0.836
**ART**	**Pre-switch**	**Post-switch**		
**Core agent**				
NNRTI	53	–	115	
PI	121	–	60	
**INSTI type**				
Dolutegravir	–	84	–	
Elvitegravir/Cobicistat	–	49	–	
Raltegravir	–	41	–	
**Backbone agent**				
TDF/FTC (Truvada)	136	94	159	
TAF/FTC	–	48	–	
ABC/3TC (Kivexa)	32	53	14	
AZT/3TC (Combivir)	32	5	27	

Data is expressed as mean (M) with standard deviation (SD) or as frequency (n) with percentage (%); *y.o. * years old; *BMI * Body mass index, *ART * antiretroviral therapy, *NNRTI * Non nucleoside reverse transcriptase inhibitors, *PI * Protease inhibitors, *INSTI * Integrase strand transfer inhibitors, *TDF/FT * Tenofovir disoproxil/Emtricitabine, *TAF/FTC * Tenofovir-Alafenamide/Emtricitabine, *ABC/3TC * Abacavir/Lamivudine, *AZT * Azidothymidine/ Lamivudine

### Weight and BMI

**Fig. 1:**
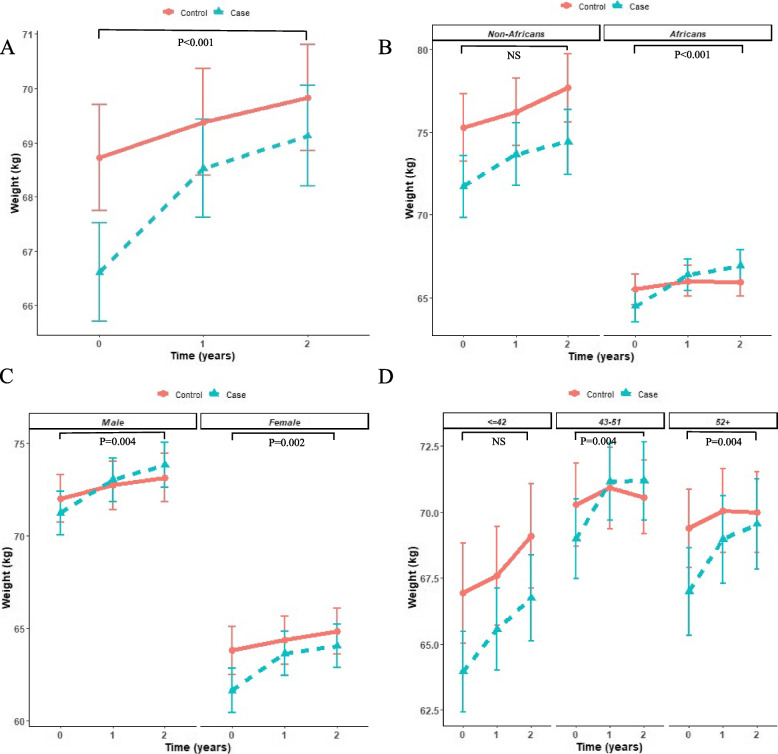
Weight gain following the switch to INSTI-based regimens. Results are presented as mean values ± standard error. Weight gain in study vs. control group (**A**), stratified by ethnicity (**B**), gender (**C**) and age (**D**). Differences in weight (kg) were calculated after 12 and 24 months of follow-up; *P*-values in each panel related to the difference between the control and study groups at 24 months; NS: not significant. Models were adjusted for CD4 and BMI at index time

Participants in both groups gained weight and their BMI increased over the 2 years of follow-up. While PLWH in the control group gained 1.10 ± 0.31 kg during the 2 years of follow-up, participants who switched to an INSTI-based regimen gained 2.51 ± 0.31 kg during the same period (*p* < 0.001). The main effect was noticed in the first year of follow-up, in which the control group gained 0.65 ± 0.27 kg, while the study group gained 1.91 ± 0.27 kg (*p* < 0.001, Fig. 1A). As expected, a similar difference was noted for BMI, as the control and the study groups BMI increased by 0.40 ± 0.12 kg/m^2^ and 0.92 ± 0.12 kg/m^2^ over 2 years, respectively (*p* < 0.001). The rate of weight gain and BMI change in the study group weakened in the second year of follow-up and became similar to the one observed in the control group (Fig. 1A).

To explore risk factors, we assessed the effect of ethnicity, gender, age, and the impact of the specific INSTI types and backbone agents prescribed.

### Ethnicity

Our cohort included 110 Caucasians and 239 African descendants. Regardless of their group, Caucasians gained significantly more weight than African descendants did (2.55 ± 0.40 vs 1.46 ± 0.27 kg, respectively, *p* < 0.009). During the first year, both ethnicities gained weight similarly in their respective study groups. However, while the African descendants moderated the rate of weight gain in the second year of follow-up, Caucasians continued to demonstrate an increased rate similar to the one observed in the first year (Fig. 1B). Weight gain patterns differed in the two groups based on ethnicity: African descendants in the study group gained 2.43 ± 0.37 kg, while in the control group they gained only 0.45 ± 0.38 kg (*p* < 0.001). In contrast, we found no difference in weight gain between the study and control group among Caucasians (2.71 ± 0.57 kg vs 2.40 ± 0.54 kg, respectively, *p* = 0.649, Fig. 1B).

### Gender

Our cohort included 196 males and 153 females. Males and females gained 1.80 ± 0.34 kg and 1.81 ± 0.30 kg, respectively (*p* = 0.985). During the 2 years of follow-up, males’ weight increased by 2.60 ± 0.44 kg in the study group and by 1.12 ± 0.41 kg in the control group (*p* = 0.004). Among females the difference was also significant: females gained 2.42 ± 0.46 and 1.06 ± 0.50 kg in the study and control groups, respectively (*p* = 0.002). The difference between genders within the study group was not significant (2.60 ± 0.44 vs. 2.42 ± 0.46 kg among males and females, respectively, *p* = 0.743 Fig. 1C).

### Age

To assess the impact of age as a risk factor for weight gain after switching to an INSTI-based regimen, we divided the participants into three age subgroups of ≤42, 43–51 and ≥ 52 years old at time 0 and compared the weight gain in the study group vs. the control group (Fig. 1D). In the youngest subgroup, patients gained weight similarly in the control and the study group (*p* = 0.271). A significant difference in weight gain between the study and the control group was observed in the two other age subgroups. While the control group gained 0.28 ± 0.56 kg in the 43–51 age subgroup and 0.61 ± 0.56 kg in the above 52 age subgroup over 2 years, a significantly higher weight gain was observed in both age subgroups within the study group (2.19 ± 0.53 kg in the 43–51 age subgroup and 2.56 ± 0.56 kg in the above 52 age subgroup (*p* = 0.004 in both comparisons, Fig. 1D)).

### INSTIs type

Participants included in the study group were switched to one of the following INSTIs: Dolutegravir (DTG, *n* = 84), Elvitegravir/Cobicistat (EVG, *n* = 49) or Raltegravir (Ral, *n* = 41, Table 1). Participants gained 2.39 ± 0.47, 3.16 ± 0.61, and 2.096 ± 0.67 kg during the 2 years of treatment with DTG, EVG, and RAL, respectively; the differences were not statistically significant.

### Backbone agent

We identified four main groups of PLWH within the study group according to the backbone drug they received before and after the switch to an INSTI-based regimen. Participants who were treated with Tenofovir disoproxil/Emtricitabine (TDF/FTC) before and after the switch (*n* = 70) gained 2.01 ± 0.51 kg; those who were treated with Abacavir/Lamivudine (ABC/3TC) before and after the switch (*n* = 24) gained 3.87 ± 0.87 kg. Participants who changed from TDF/FTC to Tenofovir-Alafenamide/Emtricitabine (TAF/FTC, *n* = 29) gained 2.91 ± 0.79 kg, and those who switched from TDF/FTC to ABC/3TC, (*n* = 16) gained 1.54 ± 1.07 kg. The differences between the groups were statistically non-significant (*p* = 0.396).

**Fig. 2:**
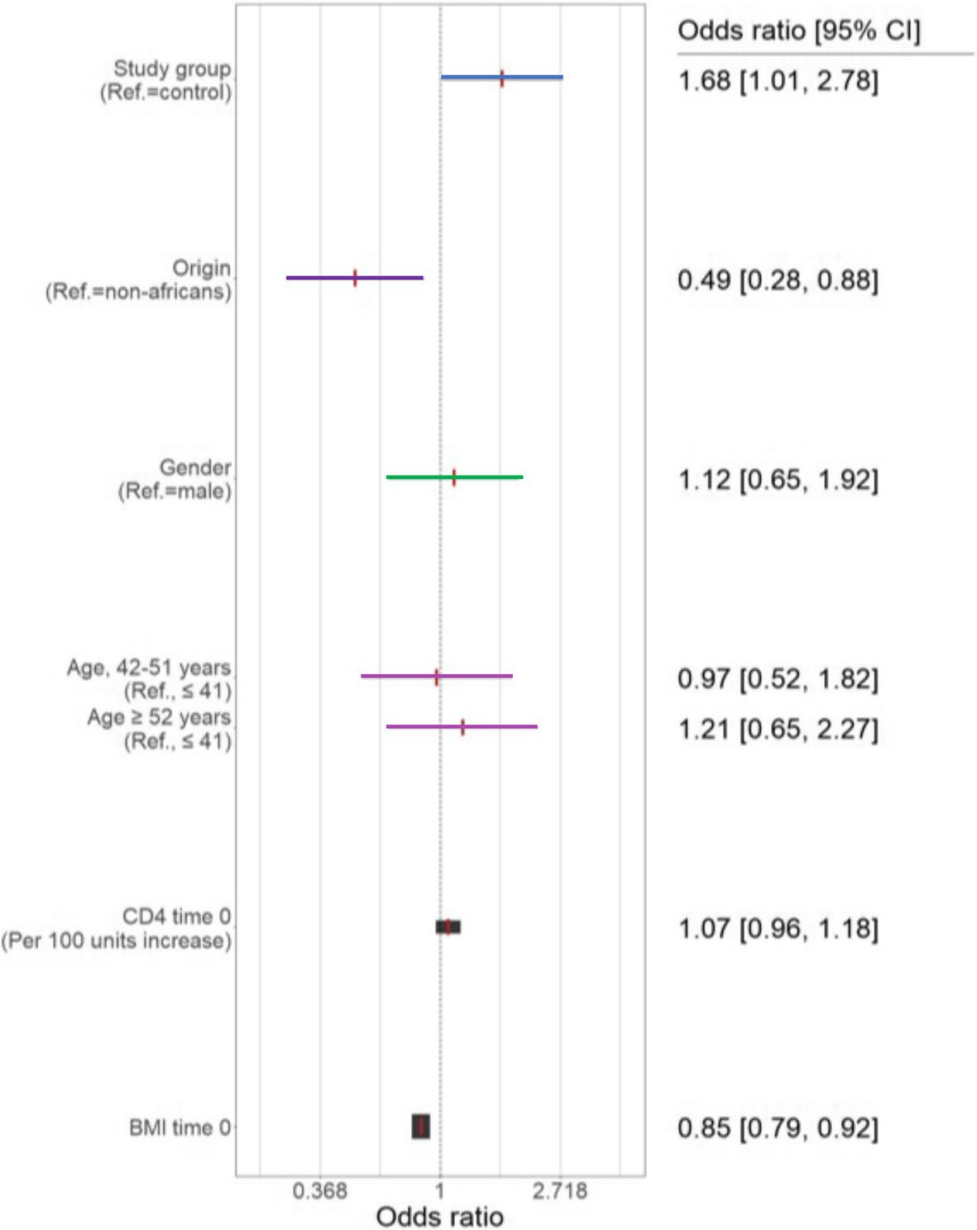
Multivariate regression analysis for weight gain of ≥5% of initial body weight during 24 months. The width of the confidence interval (CI) line is proportional to the CI (a smaller CI is expressed in a wider line)

We also performed a multivariate logistic regression analysis for ≥5% weight gain during 24 months of follow-up. Switching to INSTI treatment (the study group), Caucasian origin, and initial low BMI were found to be significant risk factors for excessive weight gain (Fig. 2). These parameters were confirmed by a sensitivity analysis of the upper quintile of the relative difference in weight either within 1 year of follow-up or 2 years. We performed the same analysis to investigate whether other risk factors for excessive weight gain are significant after switching to INSTI, including only patients from the study group. This analysis indicated higher initial BMI at the switch was associated with a smaller weight gain after the switch (OR 0.87, CI 0.79–0.96; *p* = 0.0005).

### Metabolic parameters

To explore the effect of INSTIs on parameters related to metabolic syndromes such as hyperglycemia, hyperlipidemia, and hypertension, we examined whether the groups were comparable in terms of antidiabetic medications, lipid-lowering agents, and antihypertensive treatment status during the 2 years of follow-up. The proportion of participants who were already receiving these treatments at baseline, those who initiated such treatment during follow-up and those who did not necessitate such treatment was similar between the groups (*p* = 0.678, *p* = 0.493, and *p* = 0.298 for antihypertensive, antihyperglycemic and antihyperlipidemic treatment, respectively).

### Glucose

Blood glucose levels were comparable between the two groups at baseline: 97.1 ± 28.0 mg/dL in the control group and 97.7 ± 28.2 mg/dL in the study group (*p* = 0.836). The average glucose levels in the control group were stable during the first year of follow-up (96.7 ± 28 mg/dL, *p* = 1.000), and by the end of the second year of follow-up, a slight decrease was observed (95.6 ± 25.4 mg/dL, *p* = 0.962). In contrast, the study group experienced a constant increase in glucose levels throughout the 2 years of follow-up, which reached 98.6 ± 33.8 mg/dL at the end of the follow-up period (*p* = 0.865). Generally, exploring the effect of race or gender, none of these parameters had an independent impact upon glucose levels changes. When we analyzed only the subgroup of African-origin males, there was a significantly higher increase in glucose levels in the study group (9.05 ± 3.38 mg/dL) during 2 years of follow-up after the switch compared to the control group, where glucose levels decreased by 1.6 + 3.38 mg/dL (*p* = 0.0009).

### Mean blood pressure (MPB)

There was no difference between the groups in the change of MBP (*p* = 0.522).

### Lipids’ profile

No statistically significant differences were noted between the groups regarding the measured fasting lipids: total cholesterol, HDL, LDL, and triglycerides over the two-years follow-up period. (*p* = 0.899, *p* = 0.269 *p* = 0.827 and *p* = 0.420, respectively).

## Discussion

Our study adds to the existing data on INSTI-based ART and short-term weight gain. We report that PLWH who switched to an INSTI-based regimen gained more weight over 24 months than PLWH remaining on a non-INSTI-based regimen. Most of the difference was observed during the first year of follow-up; both groups demonstrated a similar rate of weight gain after that. Our results suggest that weight gain following the switch to an INSTI-based regimen, although statistically significant, is relatively moderate and may be limited only to the first year after the switch. A direct comparison between the groups showed a higher weight gain among older patients and African descendants in the study group. However, a multivariate logistic regression analysis did not confirm these results. Instead, it indicated that lower initial weight constitutes a risk factor for a significant weight gain (≥5%) among PLWH after a switch to an INSTI- based regimen.

Previous studies performed in different populations resulted in conflicting results, with some noting weight gain [7, 10, 13–15, 22–24], which was not confirmed by others [8]. The main differences between the studies relate to the magnitude of weight gain, the impact of weight gain on the development of comorbidities, and the differential effect of specific ART combinations.

Substantial weight gain has been reported in studies of treatment-naïve PLWH. Two open-label randomized African studies assessed the effect of Dolutegravir (DTG) as compared to Efavirenz (EFV, an NNRTI) in treatment-naïve PLWH. They showed an excessive weight gain induced by DTG of 5–8 kg vs. 2–3 kg induced by EFV [11, 23]. In a retrospective observational cohort study from the USA, DTG was associated with a 6 kg increase in weight compared to 2.6 kg with NNRTI treatment; 84% of the last group were treated with EFV (*p* < 0.05) [9]. In a pooled analysis of randomized controlled studies of treatment-naïve PLWH, INSTI-based regimens resulted in a more minor but still significant weight gain of 3.24 kg, in comparison to 1.93 kg with NNRTIs (*p* < 0.001) and 1.72 kg with PIs (PIs vs. NNRTIs, *p* = 0.6) over 96 weeks [13]. A minor degree of weight gain was reported by Norwood et al. and Saber et al. [14, 24], who enrolled ART-experienced and virologically suppressed PLWH who switched to INSTI-based regimens and showed a 2.7 kg [14] and 2 kg [24] weight gain in comparison to PLWH who continued NNRTI or PI-based regimens. Recently, pooled data from randomized controlled trials reported a 1.6 kg weight gain over 48 weeks following a switch to INSTI (EVG, DTG, or Bictegravir), in comparison to a 0.4 kg weight gain in patients remaining on their previous treatment, including PI-based regimens and NNRTI-based regimens (EFV, Rilpivarin or Nevirapine), [15].

Identifying risk factors associated with weight gain in PLWH treated with INSTI is a growing research topic. Sax et al. identified INSTI initiation, low initial CD4 count, high viral load, female gender, and black race as risk factors for weight gain among over 5000 patients who initiated ART in a pooled analysis [13]. The ADVANCE trial, which almost exclusively enrolled African patients, identified similar risk factors [23]. In contrast, Bourgi et al., who identified DTG as associated with the highest weight gain in ART-naïve PLWH, did not support any other risk factors, as neither gender nor race significant effected weight gain [9]. In a switch study, Lake et al. [25] identified African ethnicity, female gender, and age > 60 as risk factors for weight gain. Erlandson used pooled data of PLWH from switch studies [15] and observed that younger age and underweight represented risk factors for weight gain greater than 10% of initial weight, which is considered clinically significant. Unfortunately, the discrepancies between the studies and the analyses, including ours, stemming from different study designs (RCTs or observational), different patient characteristics (naive, treatment experienced and diverse populations), different diets and heterogeneity of ART make it challenging to identify the patients that are prone to weight gain following a treatment switch.

Our study did not identify an association between weight gain and specific ART regimens, probably due to the high heterogeneity of the ART regimens used and the limitation of a small sample size. Previous studies showed that 2nd generation INSTI, such as DTG or Bictegravir (BIC), as well as Rilpivirine (NNRTI) or TAF/FTC (NRTIs), are associated with the most pronounced weight gain effect [9, 13, 24].

Rebeiro et al. observed a higher risk of diabetes mellitus (Hazard ratio 1.17) in patients who initiated INSTI-based ART in comparison to NNRTIs or PIs [26]. Summers et al. found a tendency to develop hyperglycemia in virally controlled women who used INSTIs [27], which was not confirmed by others [28, 29]. Recently, Millic et al. observed improved glycemic control with the use of INSTI, except for those with a weight gain of ≥5% [22]. The only metabolic effect observed in our cohort other than weight gain was an increase in glucose levels following a switch from NNRTIs or PIs to INSTI, solely in the subgroup of African descendant males. Similarly to others [30], we did not identify a worsening of other metabolic parameters such as hypertension or dyslipidemia, following the treatment switch.

The mechanism driving weight gain in this setting remains elusive, and some explanations were suggested. A rapid reduction of HIV RNA by INSTI’s correlates with the lower patients resting energy, thus causing more weight gain after treatment initiation in naive PLWH [31]. The impaired adipogenesis and adipocyte metabolism elicited by EFV occurred in higher concentrations of EVL, while RAL had neutral actions of adipogenesis [32, 33]; RAL was shown to increase zonulin, a marker of integrity of intercellular tight junctions, thus probably reducing bacterial translocation from the gut and inflammation, another driving force of weight reduction [34]. Another suggested mechanism is the interference of some INSTIs with the melanocortin signaling system, which is involved in the regulation of energy homeostasis, food intake, and satiety [35]. Variations in weight gain may also derive from unique adverse effects caused by different regimens [36]. Common side effects of PIs treatment are severe nausea and diarrhea [37], and NNRTIs have a more prominent neuro-depressant effect, that can result in depression and reduced appetite [38, 39]. This explanation could be supported by the short-term excessive weight gain noted in our study as PLWH suffer less from the side effects of PIs and NNRTIs noted after ART switch to INSTI, thus gaining excessive weight mainly shortly after the switch. It is likely that a combination of these mechanisms may explain weight gain in treated patients.

The limitations of our study include a relatively small sample size, and heterogeneity of ART medications used in both study and control groups. Furthermore, our data is retrospective; therefore, patients whose weight was not consistently recorded were excluded. Notably, the retrospective observational nature of the study does not allow to make assumptions regarding causality.

In conclusion, switching to INSTI-based antiretroviral treatment resulted in a moderate weight gain over 2 years of follow-up. This obesogenic effect mainly developed in the first year after the treatment switch. In contrast, a diabetogenic effect noted in the second year after the switch to INSTIs was observed only in a subgroup of African origin males. Although our results did not show a clinically significant weight gain following the switch to INSTI in most of the participants, the potential risk of weight gain should be discussed with patients before starting INSTI-based regimens. Both patients and their caretakers should be encouraged to closely monitor weight and long-term related metabolic features, while enhancing physical activity.

## Data Availability

All data is available and will be provided upon request from the last author, Dr. Hila Elinav, hilaelinav@gmail.com.
